# SPECIFIC COMPONENTS OF MANUAL DEXTERITY ARE AFFECTED IN PATIENTS WITH WRITER’S CRAMP: AN OBSERVATIONAL COMPARATIVE STUDY AND PRELIMINARY REHABILITATION REPORT

**DOI:** 10.2340/jrm.v58.45215

**Published:** 2026-04-09

**Authors:** Jean-Pierre BLETON, Raphael B. TAKYI, Marion VERNEAU, Cedric MONCHAUD, Thierry Peron MAGNAN, Sophie SANGLA, Amélie YAVCHITZ, Marc A. MAIER, Påvel G. LINDBERG

**Affiliations:** 1 Neurology Department, Rothschild Foundation Hospital, Paris; 2 Clinical Research Department, Rothschild Foundation Hospital, Paris, France; 3 Department of Clinical Science, Karolinska Institutet, Danderyd University Hospital, Stockholm, Sweden; 4 Université Paris Cité, Institute of Psychiatry and Neuroscience of Paris, Inserm U1266, Paris; 5 Institut de Formation en Masso-Kinésithérapie (IFMK) de l'AP-HP, Paris; 6 Institut de Formation en Masso-Kinésithérapie (IFMK), CEERRF, Saint-Denis; 7 Université Paris Cité, CNRS, Saints-Pères Paris Institute for the Neurosciences, Paris, France

**Keywords:** focal hand dystonia, handwriting, manual dexterity, rehabilitation, writer’s cramp

## Abstract

**Objective:**

To compare manual dexterity in patients with writer’s cramp and healthy controls to determine which components of dexterity are impaired in writer’s cramp. In addition, to assess the effects of rehabilitation.

**Design:**

Cross-sectional primary study, longitudinal secondary study.

**Subjects/Patients:**

23 patients with writer’s cramp and 20 healthy age- and sex-matched control subjects.

**Methods:**

Degree of manual dexterity (through 3 tasks) and handwriting symptoms were assessed, and effects of rehabilitation probed.

**Results:**

Patients with writer’s cramp showed significantly lower handwriting speed (97 letters/min) and legibility (BFM score = 1.3) compared with control subjects (171 letters/min, *p* < 0.001; BFM score = 0.15, *p* < 0.001). Only the task quantifying finger independence showed weaker finger selectivity (median = 0.84) in patients compared with controls (median = 0.89, *p* = 0.01). The other 2 tasks, maximal finger tapping and visuomotor finger force-tracking, did not reveal significant group differences (all *p* > 0.44). In patients, automated writing legibility correlated with finger selectivity (*r* = 0.50, *p* = 0.02). Rehabilitation improved dexterity (selectivity and force-tracking) and automated writing legibility.

**Conclusion:**

In this multi-component analysis of manual dexterity, reduced selectivity of finger movements was identified as the main behavioural mechanism (deficit) in writer’s cramp, associated with writing speed. In contrast, force control and tapping speed were unaffected. Selective finger activation can be improved with therapy targeting dextrous finger movements.

## LAY ABSTRACT

Although writer’s cramp is considered a handwriting-specific disorder, some deficits in wider upper-limb and hand use have previously been observed. However, for dextrous hand movements in particular, it remains unclear which components of manual dexterity are impaired in writer’s cramp and how they change with rehabilitation. We assessed these components (e.g., finger force control, finger tapping, finger independence) and found that finger independence (selective movement of 1 finger without concomitant movements of neighbouring fingers) was affected in writer’s cramp, but not the other components of dexterity. Lower finger independence was related to slower handwriting speed and worse legibility, i.e., typical symptoms of writer’s cramp. Rehabilitation improved manual dexterity, but bettered handwriting only modestly. Clinically, our results indicate that patients with writer’s cramp do show hand movement deficits in the non-writing context, and suggest that these patients can profit from a wider rehabilitation approach than conventional training of handwriting.

Writer’s cramp is considered a task-specific focal dystonia associated with handwriting (1), characterized by involuntary, repetitive, and intermittent muscle contractions resulting in abnormal postures and movements of the arm, forearm, and hand. Several clinical phenotypes have been described, and the large symptom variability poses challenges for therapeutic management (2). Often considered the most common strictly task-specific dystonia, writer’s cramp occurs predominantly during handwriting and usually disappears during other manual tasks (3). Writer’s cramp is further characterized by atypical co-contraction of antagonist muscles, resulting in impaired manual dexterity required for handwriting, and usually emerging as soon as the pen has been grasped (1, 4). However, various studies suggest that the sensorimotor alterations underlying this condition may extend beyond the strict graphomotor context: dysfunctions in upper limb and hand use have been reported during non-writing manual tasks (tapping, grasping, reaching), including excessive muscle co-contraction, bilateral sensorimotor integration deficits, and altered movement times (5–10). In terms of neural structure and function, it is thought that abnormal parieto-premotor connectivity and altered somatosensory information processing (9, 11) not only cause the specific symptoms of writer’s cramp, but also the more general non-writing motor deficits. While some authors argue for a strictly task-specific phenomenon (12), current evidence indicates that the dysfunction affects the entire upper-limb sensorimotor system, even though the primary clinical symptoms remain confined to handwriting (13).

Determining the underlying sensorimotor impairments is critical for understanding the pathophysiology and for guiding assessment and monitoring. However, behavioural studies have reported divergent impairments: several studies showed disturbed visuomotor grip force-control in patients with writer’s cramp indicating impaired sensorimotor integration (4, 10) and excessive pen grip-force during writing (14). In contrast, another study found normal pen grip-force, but perturbed writing kinematics, suggesting disturbed finger movement coordination (15). Moreover, impaired finger individuation was reported in focal hand dystonia (16) as well as in patients with musician’s dystonia (17).

Several rehabilitation approaches have been developed to improve the symptoms, such as enhanced (auditory) pen grip-force feedback during writing (18), or training of individuated finger movements (19). These studies suggest that impaired finger force control, sensorimotor integration, and individuated finger movements may constitute the behavioural mechanisms provoking writer’s cramp. However, studies characterizing these aspects of finger control required for dextrous tasks are lacking.

Assessment of manual dexterity in non-writing tasks thus represents a key objective for elucidating the symptomatology and pathophysiology of dystonia. Clinically, such a differentiation is relevant: if motor impairments are detectable in other manual tasks, treatment programmes should target dextrous sensorimotor control, whereas purely handwriting-specific deficits would corroborate therapies tailored to handwriting. Novel instrumented devices, such as the Finger Force Manipulandum (20), now enable quantified, objective assessment of key components of manual dexterity to characterize the dexterity profile of individuals with writer’s cramp. In this context, we hypothesized that patients with writer’s cramp would show deficient manual dexterity beyond handwriting, reflecting known pathophysiological mechanisms such as impaired inhibition (1). As a secondary hypothesis, we expected that rehabilitation of dexterity would provide similar improvement of handwriting as specific graphomotor rehabilitation.

## METHODS

### Study design

The primary study was cross-sectional; the secondary longitudinal, single-blinded and randomized.

### Participants

Patients with writer’s cramp (*n* = 23) were recruited at Hôpital Fondation Adolphe de Rothschild. This is a large sample size in this patient population, minimizing risk of bias and ensuring adequate sample size for comparison of multi-component dexterity measures with controls. Inclusion criteria: age 18–70 years, diagnosis of focal or segmental writer’s cramp with writing speed < 140 letters/min. Patients treated with botulinum toxin were eligible if last injection occurred > 3 months before baseline assessment. Ability to attend rehabilitation sessions. Exclusion criteria: other writing disorders (e.g., writing tremor), neurological comorbidities (e.g., Parkinsonian syndrome), recent ( < 6 months) upper limb pathology. Healthy age-, sex-, and handedness-matched control subjects (*n* = 20) were also recruited. Study procedures were approved by the Comité de Protection des Personnes Ile de France III, approval No. 3413 and Région Centre-Ouest1 de Tours, approval No. 2018T2-08. The study was registered at ClinicalTrials.gov (identifiers: NCT02882334 and NCT03797638). Informed consent was obtained from each participant prior to participation. The STROBE reporting guideline for cross-sectional studies was used to draft this manuscript. Patients’ demographic and clinical features are presented in Table I. A subset of patients (*n* = 13) initiated a rehabilitation protocol and provided longitudinal data obtained at T0 (initial assessment, pre-rehabilitation), T1 (post-rehabilitation), and T2 (follow-up: 4 ± 1 weeks post-rehabilitation). The recruitment flowchart is shown in Fig. S1.

**Table I:** Demographic and clinical characteristics of the patients with writer’s cramp (*n* = 23) and corresponding counts and averages for healthy control subjects (*n* = 20)

Subject ID	Age (years)	Sex	Dominant hand (R/L)	Occupation	Time since diagnosis (months)	Legibility BFM-score (0–4)	Speed (letters/min)	Diagnosis	Dystonic writing pattern	Rehabilitation
1	70	M	R	7	36	1	71	S.WC	Wrist, Forearm, Shoulder	GM
2	54	F	R	3	288	NA	NA	D.WC	F2, F5, Wrist, Forearm	FFM
3	51	F	L	3	10	1	73	S.WC	F2, Forearm	
4	43	F	R	5	6	2	68	S.WC	Wrist, Forearm	GM
5	57	M	R	2	204	1	111	D.WC	F1, F2, Wrist	GM
6	62	M	R	3	24	2	62	S.WC	F1–F5, Wrist, Forearm	
7	21	F	R	8	145	0	ND	S.WC	F1, F2, F5	FFM
8	58	M	R	3	13	2	90	S.WC	F1, Wrist	FFM
9	60	M	R	3	6	1	120	D.WC	F1–F5, Forearm, Shoulder	GM
10	29	F	L	3	48	0	120	S.WC	F1, F2, F3, F4	FFM
11	46	F	R	5	6	2	92	S.WC	F1, F2, F3, Wrist	
12	52	F	R	5	25	1	120	S.WC	F1, F2, Wrist, Forearm	FFM
13	61	M	R	3	1	2	111	S.WC	F1, Wrist, Forearm, Shoulder	
14	41	M	R	4	19	1	116	S.WC	F1, F2, Shoulder	FFM
15	24	F	R	3	116	1	116	S.WC	F1, F2, F3	
16	57	F	R	3	NA	1	76	D.WC	Wrist, Forearm	
17	63	M	R	3	15	2	90	D.WC	F2, Wrist, Elbow, Shoulder	GM
18	44	F	R	3	12	2	60	D.WC	F1, F2, Wrist, Forearm	
19	59	M	R	8	5	1	62	S.WC	F1, F2, F3	
20	24	F	R	8	360	1	102	S.WC	F1–F5, Wrist	
21	62	M	R	3	5	2	62	S.WC	F1–F5, Wrist, Forearm	FFM
22	40	F	R	5	7	1	125	D.WC	F1–F5, Forearm	
23	68	F	R	7	24	1	139	D.WC	F1–F5	FFM
	Average (or count) over *n* = 23 patients									
1–23	50	10M	21R	4.4	62	1.3	95	14 S.		
	Average (or count) for *n* = 20 healthy control subjects									
1–20	45	9M	18R	5.5		0.15	172			

Professional occupation: according to 8 categories of the Institut National de la Statistique et des Études Économiques (Insee, Table SI); Time since first diagnosis, Type of writer’s cramp diagnosis: classified clinically (28) as simple writer’s cramp (S.WC, only handwriting affected) or dystonic writer’s cramp (D.WC, one or more manual tasks affected in activities other than writing); The One-Minute Writing Speed Test (21) was used to assess handwriting speed (letters/min); Legibility according to the Burke-Fahn-Marsden dystonia disability scale (BFMDRS-D), (22): 0 = Normal, 1 = Slightly abnormal but legible, 2 = Moderately abnormal; legible with effort, 3 = Severely abnormal; illegible without great effort, 4 = Unable to write; Dystonic writing pattern (assessed during the handwriting test): gives upper limb localization of abnormal dystonic posture; Type of rehabilitation: GM: graphomotor training; FFM: FFM dexterity training; Abbreviations: M: male; F: female; R: right; L: left; F1: thumb; F2: index finger; F3: middle finger; F4: ring finger; F5: little finger; NA: not available = missing data; FFM: Finger Force Manipulandum.

### Clinical assessments

**Fig. 1:**
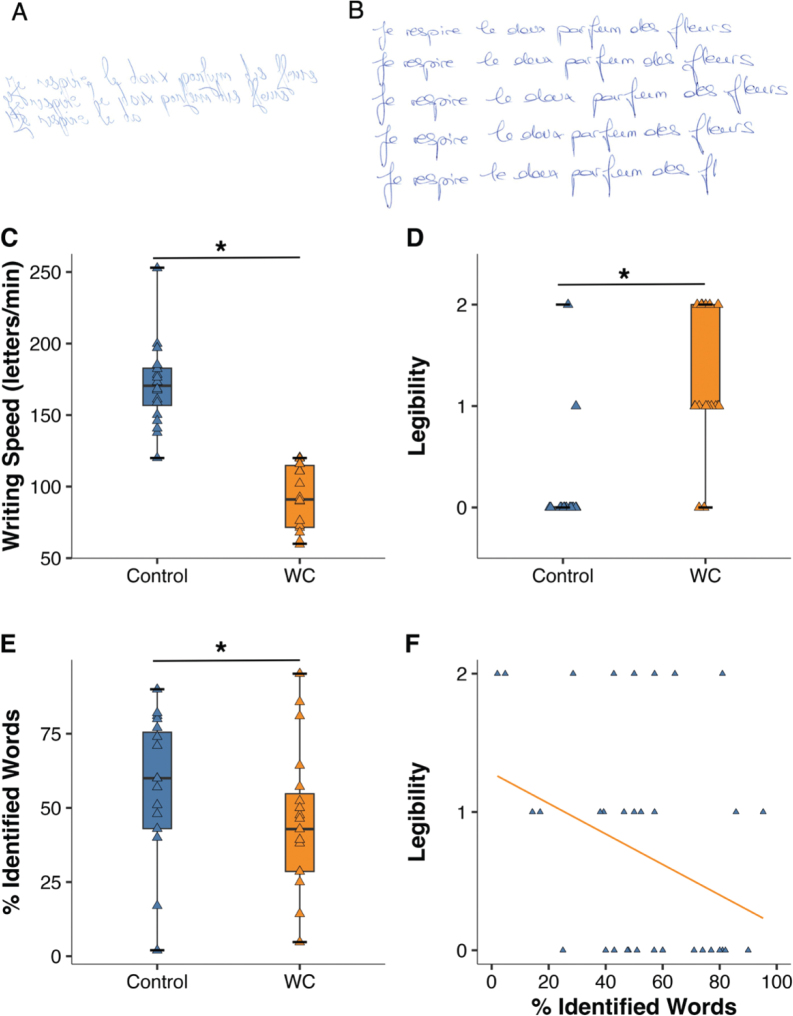
Examples and group differences in handwriting performance. (A) Handwriting example of a patient with writer’s cramp. Writing speed = 73 letters/min. Clinical legibility (BFM score = 1), Automated legibility = 14% words correctly identified. (B) Example from a healthy control subject. Writing speed = 146 letters/min. Clinical legibility (BFM score = 0), Automated legibility = 60%. (C–F) Group differences in handwriting performance (control vs writer’s cramp, WC). Box plots of (C) Writing speed, (D) Clinical legibility (BFM score), (E) Automated legibility. All 3 measures showed significant group differences (horizontal lines with asterisk; Mann–Whitney *U* test, *p* < 0.05). (F) Correlation of clinical vs automated legibility (Rho = -0.34, *p* = 0.05).

*Standard clinical postural and functional assessments*. The One-Minute Writing Speed Test was employed to assess handwriting speed (21) and the Burke-Fahn-Marsden (BFMDRS-D) dystonia disability scale (22) to code handwriting legibility (blinded rater). The handwriting samples (Fig. 1A and 1B) were also subjected to optical character recognition for automated writing-to-text conversion, implemented in OneNote software (Version 2016, Microsoft Office 365; Microsoft Corp, Redmond, WA, USA) (23). This machine learning algorithm thus provided a complementary, objective measure of legibility (% handwritten words correctly converted), shown to be clinically suitable (15).

*Rehabilitation.* Two intensity-matched rehabilitation protocols were applied: graphomotor rehabilitation or FFM (dexterity) training.

Five patients (see Table I) underwent graphomotor rehabilitation according to previous protocols based on principles of sensorimotor reprogramming and learning (24, 25). The intervention consisted of 12 sessions of 45 min each, over a period of 6–8 weeks. Sessions were divided into 2 phases of equal duration under the supervision of a therapist. The first phase helped participants to relax their upper limbs while specifically activating muscles that were not recognized as dystonic. Through finger coordination exercises and work on shoulder and wrist stability, subjects developed a control strategy to perform handwriting movements without triggering abnormal tension. The second phase consisted in decomposing the act of writing into controlled motor sequences holding a pencil. Writing was reduced to basic shapes (straight lines, loops, and arcs), and was executed slowly and with large movements in order to enhance visual and proprioceptive feedback and awareness. The focus was on reducing grip pressure and transferring effort from the extrinsic to the intrinsic finger muscles in order to limit co-contractions and promote wrist and forearm relaxation. Handwriting was gradually reintroduced by tracing simple graphic patterns, always with minimal effort and fluid execution, in order to exclude dystonic muscles from the writing movement (26).

Eight patients (see Table I) underwent FFM training, focusing on force control and finger individuation. A session consisted of 2 tasks: force-tracking, with the index and then the middle finger (total = 96 trials), and multi-finger tapping (total = 128 taps, see below).

**Fig. 2:**
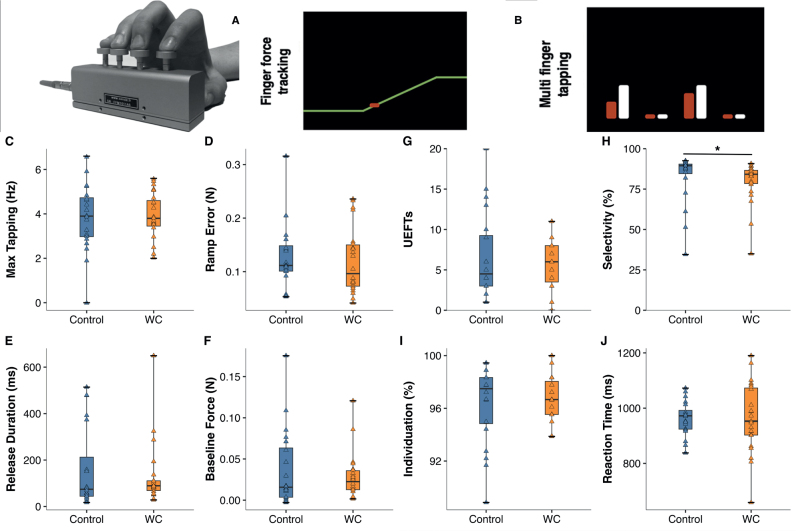
Group differences in FFM performance at T0 = baseline. Top: Finger Force manipulandum (FFM) and (A) screen display of force-tracking task (red: cursor; yellow: right-to-left moving target line). (B) Display of MFT task (white bars: indicate a target tap: here simultaneous index and ring finger tap; red bars: taps (force) applied on correct pistons). (C–F) Box plots comparing performance of control vs writer’s cramp (WC) group in (C) Max. tapping speed, and (D–F) in force-tracking. (D) Tracking error during the cramp, (E) Release duration, (F) Baseline force. None showed significant group differences. (G–J) MFT task performance. (G) Unwanted extra-finger taps (UEFTs), (H) Finger selectivity, significantly smaller in WC (Mann–Whitney *U* test, *p* = 0.01), (I) Finger individuation, (J) Reaction time.

*Dexterity.* Manual dexterity is a multi-dimensional construct, and we have developed the Finger Force Manipulandum (FFM, Fig. 2A) to extract and quantify the key components of dexterity (20). Here we implemented 3 separate visuomotor FFM tasks: *(i*) maximal tapping rate, (*ii*) force-tracking, (*iii*) multi-finger tapping. Briefly (20), the *maximal tapping rate* task consisted in repetitive tapping as fast as possible with the index finger for 10 s. The *force-tracking* task consisted in varying the force on the index finger piston to control a cursor on the computer screen in real-time (see Fig. 2A). Subjects were instructed to follow the target force as closely as possible with the cursor. The target force (a line) passed from right to left over the screen, presenting successive ramp-hold-and-release trials. Each trial consisted of a ramp phase (linearly increasing force over 1.5 s), a hold phase (constant force for 4 s) and a release phase (instantaneous return to resting force level, 0 N), followed by a resting phase (2 s). The 24 successive trials were organized in 4 blocks of 6 trials, 2 blocks with target force of 1 N and 2 at 2 N, i.e., forces typically applied in dextrous manipulation (27). Task duration was 200 s. An identical session was also performed with the middle finger. In the *multi-finger tapping* (MFT) task, subjects were instructed to reproduce 11 different finger taps according to each visual cue (Fig. 2B). The 11 different tap configurations consisted of 4 one-finger taps (of the index, middle, ring, or little finger), 6 two-finger configurations (simultaneous index–middle, index–ring, index–little, middle–ring, middle–little, or ring–little finger taps), and 1 four-finger tap, presented in pseudo-randomized order. All configurations were performed twice resulting in a total of 32 one-finger, 30 two-finger, and 2 four-finger taps. Performance measures were calculated for one-finger and two-finger tap configurations. The entire task with its 64 trials lasted about 5 min.

*FFM performance measures*. (A) Maximal index finger tapping rate (in Hz). (B) In force-tracking, we computed several measures trial-by-trial: (*i*) Root-mean-square tracking error (RMSE) between the applied and the target force, separately extracted for the ramp and hold phase. (*ii*) Release duration, computed as the time taken to reduce the force from 75% to 25% of the target force. (*iii*) Mean force during the hold, i.e., average force across 3 s excluding the first and last 500 ms of the hold. (*iv*) Mean baseline force (supposed to be = 0 N), calculated as the mean force during the resting phase between each trial from 1500 ms to 500 ms before ramp onset. (C) Multi-finger tapping (MFT) performance was also expressed by multiple measures: (*i*) Success (%), i.e., proportion of correct target (single- or two-finger) taps across all trials (not constrained to first finger tap), independent of presence/absence of UEFT; (*ii*) Number of unwanted extra-finger-taps (UEFT), i.e., ratio of trials in which other finger(s) than the target finger(s) were actively moved over total number of trials; (*iii*) Finger selectivity: % correctly achieved taps among all target taps (calculated only for the first tap, if several taps occurred in the same trial, i.e., later UEFT not counted); (*iv*) Finger individuation (%), i.e., the proportion of taps where the response did not include UEFTs, range (0–100%); and (*v*) Reaction time (ms).

### Statistical analysis

Shapiro–Wilk tests were used to check for normally distributed (given as mean±SD) or skewed performance measures (median [IQR]). Maximal tapping rate was normally distributed, but all performance measures of Line-tracking, and of MFT, except reaction time, were significantly skewed. For reasons of consistency in analysis, all FFM performance and clinical measures were treated as median [IQR]. Mann–Whitney *U* test was used for assessing group differences in central tendency. Longitudinal data were analysed using within-group Friedman ANOVA across the 3 time-points (T0, T1, T2), including *post-hoc* Wilcoxon matched pairs testing. Relations between 2 variables were explored with Spearman rank correlations.

## RESULTS

### Baseline (T0) group comparisons in handwriting

Fig. 1A shows handwriting examples of a patient and Fig. 1B those of a control subject. The 30-letter French sentence to be written repetitively for 1 minute was “Je respire le doux parfum des fleurs” (“I smell the sweet scent of flowers”). Compared with control subjects (median 171 letters/min [IQR 154 to 184]) patients with WC showed significantly slower handwriting speed (91 letters/min [72 to 114], *p* < 0.001, Fig. 1C), as well as significantly worse clinical legibility (BFM score: controls 0.0 [0 to 0] vs WC 1.0 [1 to 2], *p* < 0.001, Fig. 1D) and automated word legibility (controls 60% identified words [43 to 77] vs WC 43% [29 to 57], *p* = 0.048, Fig. 1E). Clinical and automated legibility correlated (Rho = –0.34, *p* = 0.05, Fig. 1F).

### Baseline (T0) group comparisons in FFM tasks

*Maximal tapping rate*. There was no group difference in tapping speed (controls median 3.9 Hz [IQR 3.0 to 4.8] vs WC median 3.8 Hz [IQR 3.4 to 3.7], *p* = 0.69; Fig. 2C). Neither was there any significant group differences in *Finger force tracking*: not in tracking error during the ramp (controls median 0.11 N [0.1 to 0.15] vs WC median 0.09 N [0.07 to 0.15], *p* = 0.49 Fig. 2D); not in release duration (controls median 74 ms [42 to 266] vs WC median 89 ms [65 to 111], *p* = 0.44, Fig. 2E), nor in baseline force (controls median 0.02 N [0.004 to 0.07] vs WC median 0.02 N [0.01 to 0.04], *p* = 0.70, Fig. 2F). In *Multi-finger tapping* there was no group difference in success rate (controls median 84 % [79 to 90] vs WC median 84% [76 to 89], *p* = 0.89), neither in UEFT (controls median 4.5 [3 to 9.5] vs WC median 6.0 [3 to 8], *p* = 0.99, Fig. 2G), nor in individuation (controls median 97 % [95 to 98] vs WC median 97 % [96 to 98], *p* = 0.92, Fig. 2I) or in reaction time (controls 972 ms [923 to 999] vs WC 952 ms [902 to 1077], *p* = 0.99, Fig. 2J). But there was a significant group difference in finger selectivity (controls median 89 % [84 to 91] vs WC median 84 % [78 to 87], *p* = 0.01, Fig. 2H).

### Baseline (T0) correlations between handwriting and FFM task performance

**Fig. 3:**
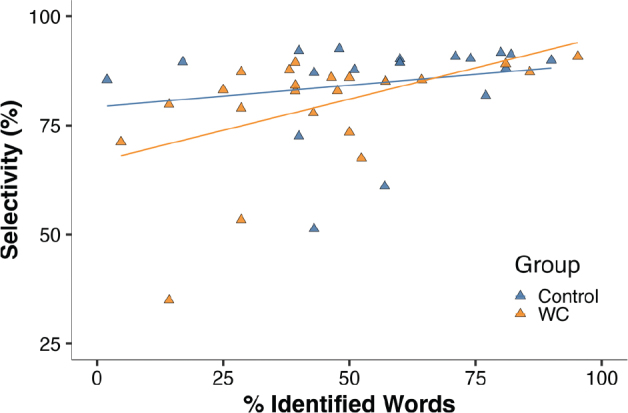
Correlation between automated handwriting legibility and finger independence in the MFT task. Percentage correctly identified words (legibility) correlated significantly with FFM finger selectivity (orange-coloured symbols/regression line, Rho = 0.49, *p* = 0.02) in patients with writer’s cramp (WC), but not in control subjects (blue symbols, Rho = 0.2, *p* = 0.41).

We limited correlation testing to dexterity variables found to be different from control subjects. In patients with writer’s cramp there was a correlation between quality of handwriting and performance on the MFT finger individuation task (Fig. 3): good finger selectivity correlated significantly and positively with better legibility (automated legibility measure, Rho = 0.49, *p* = 0.02). This was not the case in control subjects (Rho = 0.30, *p* = 0.21).

### Descriptive longitudinal changes with rehabilitation

**Fig. 4:**
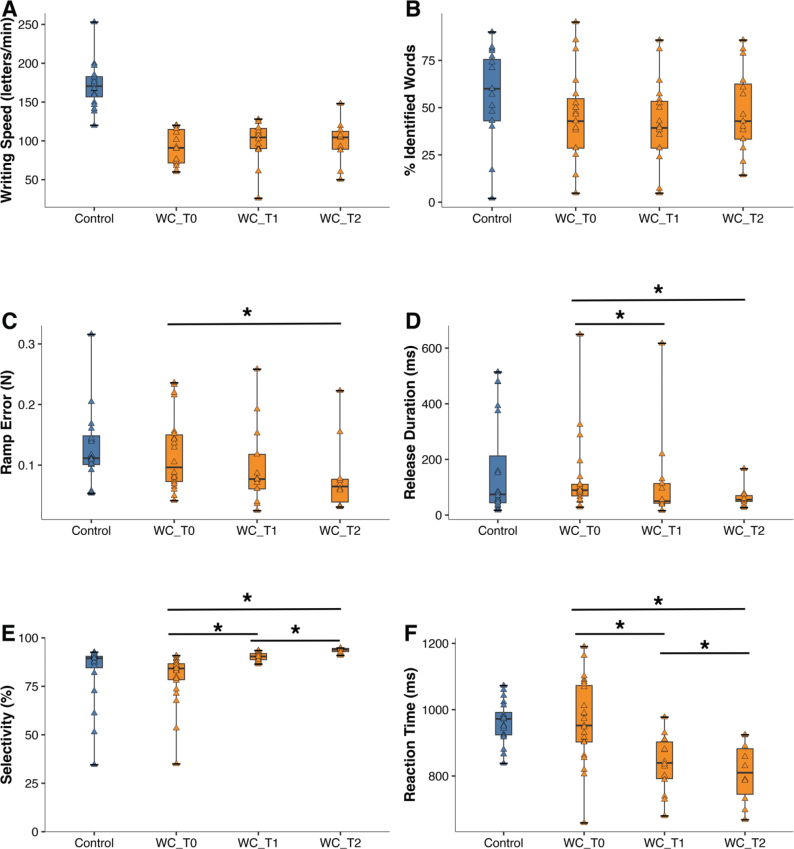
Rehabilitation: longitudinal changes (from T0 to T1 and T2). (A–B) Changes in handwriting quality, and (C–F) changes in FFM dexterity measures for patients with writer’s cramp (WC, orange-coloured). For comparison (blue): performance of control subjects at T0. (A) Writing speed, (B) Automated legibility (% correctly identified words), (C) Force-tracking ramp error, (D) Release duration, (E) MFT finger selectivity, (F) MFT reaction time. Horizontal lines with asterisk: significant longitudinal performance differences (*post-hoc* Mann–Whitney *U* test, *p* < 0.05; see Table II for corresponding numerical values).

A subset of patients with writer’s cramp underwent treatment (12 sessions), either graphomotor rehabilitation or FFM (dexterity) training. There was no between-treatment-group difference in outcome: both showed similar writing speed and no change of speed with therapy (FFM group: T0 pre-rehabilitation = 116 letters/min, T1 post-rehabilitation = 107, T2 follow-up = 114; graphomotor group: T0 = 111, T1 = 102, T2 = 105). Automated legibility showed some non-significant change in the FFM group (T0 = 39% correctly identified words, T1 = 50%, T2 = 42%) and remained stable in the graphomotor group (T0 = 29%, T1 = 29%, T2 = 29%). Given the small sample size and lack of change in primary outcome (writing speed), the data of the 2 treatment groups were pooled. Fig. 4 shows the changes induced by rehabilitation in writing quality and in FFM performance for the pooled data (*n* = 10, see Table II for corresponding numerical values). Handwriting speed did not improve over the 3 time points (Fig. 4A, *p* = 0.38), neither did clinical legibility, but automated legibility did improve (Fig. 4B, *p* = 0.03) with a non-significant *post-hoc* trend for increase from T1 to T2 (*p* = 0.08). Rehabilitation did not affect FFM maximal tapping rate from T0 (rate = 3.9 Hz) to T1 (4.0 Hz) and then to T2 (4.3 Hz, *p* = 0.39), but did improve force-tracking and MFT performance. Force-tracking improved on 3 performance measures: through significantly decreased tracking error (Fig. 4C, *p* = 0.03) with significant *post-hoc* change from T0 to T2 (*p* = 0.01) and trend from T1 to T2 (*p* = 0.06). Second, force-tracking also showed shorter release duration (Fig. 4D, *p* = 0.02) with significant post-hoc change between T0 to T1 (*p* = 0.006) and T0 to T2 (*p* = 0.007). And third, baseline force during tracking also decreased over time (*p* = 0.02) with significant post-hoc change from T0 to T2 (*p* = 0.046) and T1 to T2 (*p* = 0.01). Moreover, MFT performance also improved through better finger selectivity (Fig. 4E, *p* = 0.0001) with significant *post-hoc* change from T0 to T1 (*p* < 0.001), T0 to T2 (*p* = 0.005), and T1 to T2 (*p* = 0.005). And MFT reaction time became shorter over time as well (Fig. 4F, *p* = 0.001), with significant *post-hoc* change from T0 to T1 (*p* = 0.01), T0 to T2 (*p* = 0.02), and T1 to T2 (*p* = 0.005).

**Table II:** Longitudinal data: effects of rehabilitation on handwriting quality and manual dexterity (FFM task performance)

Task	Performance variable	T0 (pre-rehab, *n* = 10)	T1 (post-rehab, *n* = 10)	T2 (follow-up, *n* = 10)	ANOVA *p*-value
Handwriting	Speed (letters/min)	105 [90 to 116]	96 [88 to 110]	105 [88 to 114]	0.38
	Clin. legibility (BFM score, 0–4)	1.0 [1 to 2]	1.5 [1 to 2]	2.0 [1 to 2]	0.20
	Automated legibility (% id words)	34 [25 to 57]	33 [7 to 52]	39 [29 to 46]	0.03*
FFM Max tapping speed	Max rate (Hz)	3.7 [3.0 to 5.1]	4.0 [2.6 to 4.6]	3.9 [3.8 to 5.2]	0.39
FFM Force-tracking	Tracking error (ramp, *n*)	0.12 [0.08 to 0.15]	0.08 [0.04 to 0.12]	0.06 [0.03 to 0.08]	0.03*
	Release duration (ms)	110 [82 to 287]	53 [45 to 129]	55 [48 to 74]	0.02*
	Baseline force (N)	0.03 [0.02 to 0.04]	0.02 [0.01 to 0.03]	0.00 [0.01 to 0.02]	0.02*
FFM Multi-finger tapping (MFT)	Success rate (%)	84 [75 to 94]	92 [86 to 100]	96 [84 to 98]	0.46
	Selectivity (0–1)	0.81 [0.71 to 0.85]	0.89 [0.88 to 0.92]	0.94 [0.93 to 0.95]	0.0001*
	Reaction time (ms)	959 [862 to 1077]	861 [789 to 910]	810 [731 to 890]	0.001*

Values given as median [Q1 to Q3 range]. The *p*-value of the Friedman ANOVA pertains to the *n* = 10 patients who completed the T0, T1, and T2 measurements (of the initial 13 patients included in rehabilitation, 3 dropped out at T2, hence *n* = 10).

* Significant longitudinal change over time (from T0, over T1, to T2) at *p* < 0.05.

## DISCUSSION

To what degree writer’s cramp is clinically specific to handwriting (2, 3), or whether it reflects a wider sensorimotor impairment also expressed in non-writing tasks (13), is still debated. Here we showed that manual dexterity, in a non-writing context, is impaired in patients with writer’s cramp, but in a non-uniform manner: key components of manual dexterity (see points i–iii below) were differentially affected.

(*i*) *Finger force-tracking*. Patients with writer’s cramp showed similar precision in force control (tracking error), similar motor inhibition (release duration), and similar resting force compared with control subjects. Previous studies showed clearly higher pen grip-force during writing (14), but they reported less consistent results on force control in non-writing tasks: lifting and cyclic movements were not affected (12), but power-grip-force control was deficient, e.g., higher tracking error (4, 10) and longer release duration (4). This suggests, first, that visuomotor control of finger forces maybe less affected than power-grip forces, these latter requiring activation of more proximal arm muscles shown to be more strongly affected in writer’s cramp than distal muscles (5). Second, the type of modality involved in sensorimotor control may play a differential role: deficient sensory integration has been shown in dystonia (29), impacting somatosensory (30) and in particular visual integration (10, 31) relevant for (often deficient) visuomotor tracking in writer’s cramp (4, 10).

(*ii*) *Finger tapping speed*. This measure of single-finger movement control was not affected. This was unexpected as co-contraction of antagonist muscles, deleterious to fast tapping requiring alternating antagonist activation, has typically been observed in writer’s cramp (16). However, motor control of single finger taps may be simpler in terms of motor commands than that of pen strokes, the latter requiring muscular co-contraction for maintaining the pen grip, superposed with alternating activation of antagonists for carrying out successive pen-strokes (16).

(*iii*) *Finger individuation*. We found decreased finger selectivity in writer’s cramp, i.e., a lower degree of finger independence, which represents a key component of manual dexterity. Moreover, in patients, lower finger selectivity correlated positively with poorer (automated) handwriting legibility. A low degree of independent finger movements was recognized early in writer’s cramp (16) and is a consequence of abnormal peripheral motor commands, expressed by excessive muscular co-contraction (1, 4) and higher intermuscular coherence (32, 33), as well as in stronger cortico-muscular coherence (34). This altered command is most likely a corollary of impaired cortical inhibition, including deficient short-interval intracortical inhibition as well as reduced motor cortical surround inhibition, required for refined, specific motor output acting on the selected finger, while inhibiting the non-selected, neighbouring fingers (2, 35).

The emergence of dystonic symptoms independent of writing as shown here is coherent with pre-existing abnormal cortical and subcortical functioning, whose clinical expression is initially revealed through handwriting, although other fine motor skills also are vulnerable, such as finger selectivity, conveying a generalized disorder of fine distal sensorimotor control. The observed correlation between finger selectivity and handwriting legibility confirms the functional link between these 2 contextual domains of hand use, also mirrored in musicians with focal dystonia, in whom impaired finger individuation affects musical performance (16).

Our longitudinal rehabilitation protocol revealed only moderate improvements in handwriting. Pooled results of graphomotor and dexterity (FFM) training subgroups showed improved automated handwriting legibility, but unaffected writing speed and clinical legibility, consistent with previous data showing minor improvements after motor training in focal hand dystonia (24). Graphomotor therapy gave similar results to FFM training, suggesting that dextrous FFM training gains can generalize to writing. Moreover, both training groups showed improved finger independence. Several earlier studies have shown that the typical symptoms of writer’s cramp are partially reversible through tailored motor training, within the context of handwriting or through non-writing exercises focused on finger individuation (8, 19, 24, 36, 37), and effects can be long-lasting, although handwriting performance typically remains below pre-morbid functional levels (38, 39). This is comparable to the outcome of our rehabilitation protocol showing modest improvements. However, because motor/handwriting outcome measures varied considerably across studies (between subjective criteria, writing speed and legibility, clinical scales, and pen/grip force measures), direct comparison of treatment effects and treatment factors, such as type of rehabilitation (graphomotor vs dexterity training) and training intensity, is problematic (40).

Moreover, novel therapeutic approaches combine behavioural interventions with adjunct brain stimulation, such as low-frequency repetitive somatosensory stimulation (41), repetitive transcranial magnetic stimulation (42, 43), as well as transcranial direct current stimulation (tDCS) (43), or deep brain stimulation (44), but their clinical relevance remains to be established (45). Similarly, botulinum toxin injection, on its own (46) or as adjunct therapy (21, 47, 48), seems to be promising, but whether efficacy is better than motor rehabilitation alone remains to be established.

Whereas rehabilitation of dexterity may be considered a bottom-up approach, targeting sensorimotor components of manual dexterity (i.e., finger individuation, temporal precision, and force modulation), graphomotor rehabilitation, contrarily, favours a top-down approach, focusing on functional movement rehabilitation based on contextualized manual writing tasks, relying on controlled repetitions of visuomotor writing movements. Rehabilitation of dexterity intends to promote the re-emergence of intracortical inhibition and the differentiation of cortical somatotopic maps (36, 37), supposed to reduce pathological co-contractions and thus improve writing dynamics and kinematics indirectly. In contrast, graphomotor rehabilitation directly aims at the graphic gesture and at mobilizing cortical and cerebellar networks involved in the sensorimotor control of handwriting (17), thereby restructuring motor synergies in their functional context, with collateral effects on finger selectivity. Thus, these 2 distinct rehabilitation approaches seem to act in a partially overlapping and partially complementary manner, suggesting that combining top-down and bottom-up training approaches may improve rehabilitation efficacy (2), all the more so as they presumably involve overlapping cerebello-cortical and cortico-striatal networks (8, 49). It has indeed been shown that sensorimotor rehabilitation that improved handwriting fluency also acted on cortical functioning by reorganizing the somatotopic hand representation (2). Thus, specific graphomotor and non-specific dexterity rehabilitation appear to converge on common behavioural and pathophysiological targets, i.e., the loss of finger selectivity, by activating adaptive cortico-striatal and cortico-cerebellar plasticity, thereby restoring focal inhibitory processes required for dextrous motor control of the hand (2, 35, 37). These therapy-induced changes in pathophysiological functioning presumably lead to (somewhat) improved handwriting legibility persisting beyond the rehabilitation period, as shown by our study.

### Study limitations

The sample size of our cross-sectional investigation was in a range typical of studies on focal hand dystonia, but it was small in the longitudinal training study, thus the clinical relevance of rehabilitation needs be confirmed. Patients were not assessed for specific upper limb somatosensory deficits or other clinical sensorimotor measures, ruling out evaluation of potential somatosensory deficits and their interaction in writer’s cramp. In addition, this excluded potential cross-validation of our results through the use of complementary clinical measures. The FFM tasks did not involve the thumb, although dystonic thumb movements were observed in a majority of our patients (see Table I). Nonetheless, most patients with dystonic thumb movements also showed dystonic movements of other digits, in particular of the index finger, which was tested by the FFM tasks. The FFM may not capture all aspects and requirements relevant to handwriting (e.g., control of muscular co-contraction), thus further behavioural differences in dextrous manual control between patients and control subjects may have been missed. Nonetheless, this does not bear upon the findings of unaffected finger force control, and deficient finger independence (finger selectivity) in writer’s cramp.

In conclusion, the major finding concerns the specific deficit in finger selection as the key component of manual dexterity affected in writer’s cramp. Other components, such as force control and movement speed, were not affected. Thus, writer’s cramp was not associated with a general loss of manual dexterity. Finger selectivity was correlated to legibility, emphasizing the relevance of finger independence in handwriting. Targeted therapy, whether graphomotor or writing-independent dexterity training, improved finger selectivity, and, modestly, handwriting legibility.

## Supplementary Material




