# Supplementary material for: SPECIFIC COMPONENTS OF MANUAL DEXTERITY ARE AFFECTED IN PATIENTS WITH WRITER’S CRAMP: AN OBSERVATIONAL COMPARATIVE STUDY AND PRELIMINARY REHABILITATION REPORT
Source: J Rehabil Med. 2026 Apr 9;58:45215. doi: 10.2340/jrm.v58.45215 (PMC13071796; doi:10.2340/jrm.v58.45215)
Supplement: Supplementary file 1 [file JRM-58-45215-s1.pdf]

Table SI. French socio-professional categories according to the Institut national de la statistique et des études économiques (Insee).

Code	Category
1	Farmers and agricultural operators
2	Skilled tradespeople, shopkeepers and business owners
3	Senior managers and higher professional occupations
4	Intermediate occupations
5	Administrative and service employees
6	Manual workers
7	Retired persons
8	Other inactive persons (students, unemployed, homemakers)

From: Amossé T. Revisiting the History of Socio-professional Classification in France. *Annales (English ed)* 2013; 68: 697-732. doi:10.1017/S2398568200000157
