# Supplementary material for: SPECIFIC COMPONENTS OF MANUAL DEXTERITY ARE AFFECTED IN PATIENTS WITH WRITER’S CRAMP: AN OBSERVATIONAL COMPARATIVE STUDY AND PRELIMINARY REHABILITATION REPORT
Source: J Rehabil Med. 2026 Apr 9;58:45215. doi: 10.2340/jrm.v58.45215 (PMC13071796; doi:10.2340/jrm.v58.45215)
Supplement: Supplementary file 2 [file JRM-58-45215-s2.pdf]

Fig. S1. Recruitment flow-chart.

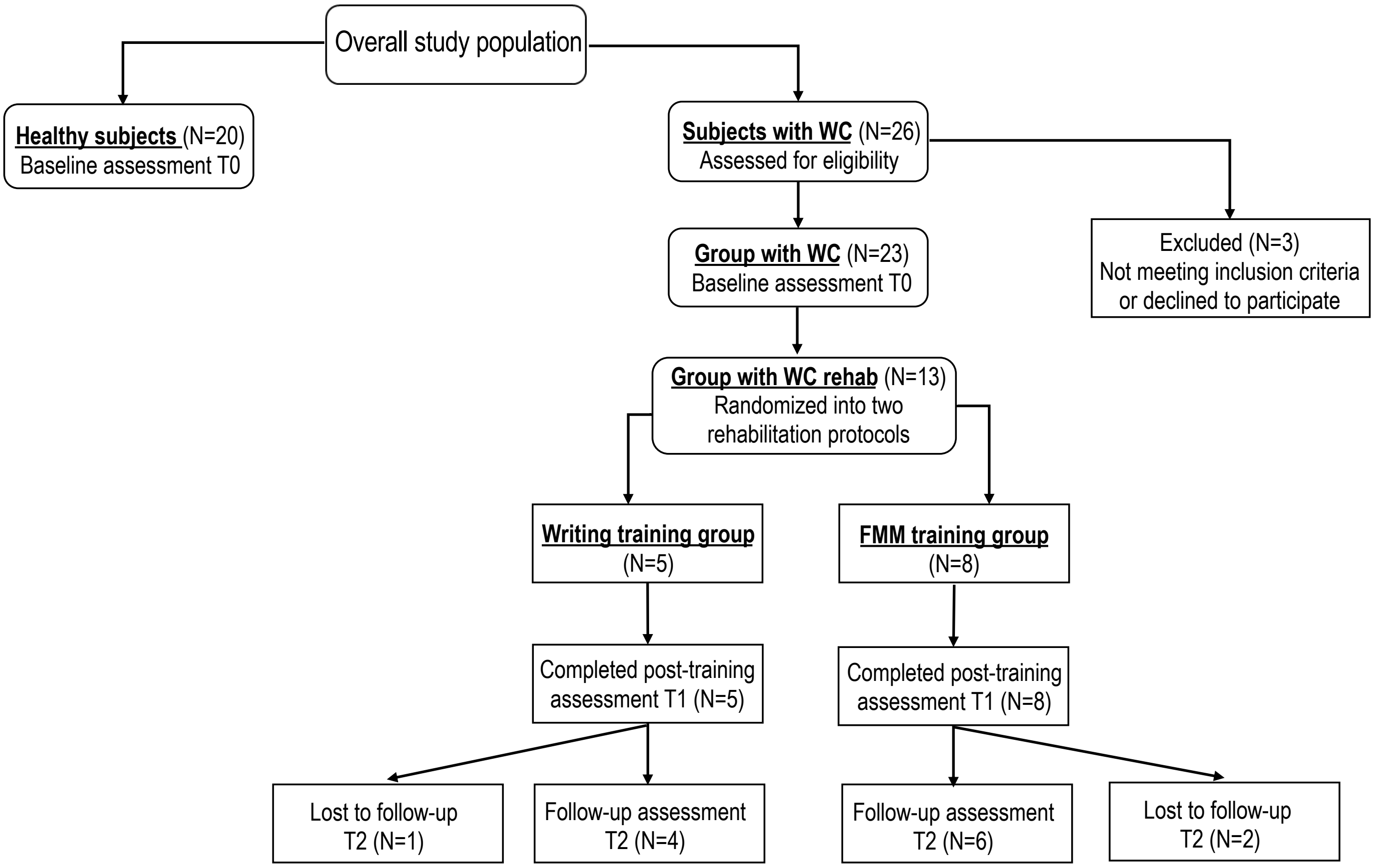

The cross-sectional study was based on N=20 healthy subjects and N=23 patients with writer's cramp, all assessed at T0. The secondary rehabilitation study concerned N=13 patients, with N=5 in the writing group, and N=8 in the FFM group. Abbreviations. WC: writer's cramp; T0: initial assessment (pre-rehabilitation); T1: assessment post-rehabilitation; T2: follow-up assessment (4±1 weeks after T1).
